# Profiling of *Leptospira interrogans*, *L. santarosai*, *L. meyeri* and *L. borgpetersenii* by SE-AFLP, PFGE and susceptibility testing—a continuous attempt at species and serovar differentiation

**DOI:** 10.1038/emi.2016.16

**Published:** 2016-03-09

**Authors:** Luisa Z Moreno, Fabiana Miraglia, Walter Lilenbaum, José SF Neto, Julio C Freitas, Zenaide M Morais, Rudy A Hartskeerl, Barbara LP da Costa, Silvio A Vasconcellos, Andrea M Moreno

**Affiliations:** 1 Laboratory of Molecular Epidemiology and Antimicrobial Resistance/Laboratory of Bacterial Zoonosis, School of Veterinary Medicine and Animal Science, University of São Paulo, São Paulo/SP 05508 270, Brazil; 2 Veterinary Bacteriology Laboratory, Department of Microbiology and Parasitology, Fluminense Federal University, Hernani Mello, 101 Niterói/RJ 24210 130, Brazil; 3 Laboratory of Animal Leptospirosis, Londrina State University, Rod Celso Garcia Cid, Londrina/PR 86057 970, Brazil; 4 WHO/FAO/OIE and National Leptospirosis Reference Centre, KIT Biomedical Research, Royal Tropical Institute, Amsterdam 391105, Netherlands

**Keywords:** leptospira, MIC, PFGE, SE-AFLP, sequencing, serotyping

## Abstract

Leptospirosis is a widespread systemic zoonosis, considered as reemerging in certain developing countries. Although the cross agglutinin absorption test is still considered the standard method for *Leptospira* identification, it presents several disadvantages. The aim of this study was to characterize *Leptospira* spp. isolated from various hosts by genotyping and broth microdilution susceptibility testing in an attempt to differentiate *Leptospira* species, serogroups and serovars. Forty-seven isolates were studied. They were previously serotyped, and species confirmation was performed by 16S rRNA sequencing. Single-enzyme amplified fragment length polymorphism (SE-AFLP) and pulsed-field gel electrophoresis (PFGE) analysis enabled the distinction of *L. interrogans* from *L. santarosai*, *L. meyeri* and *L. borgpetersenii* in two main clusters. Among *L. interrogans*, it was possible to differentiate into two new clusters the serogroup Icterohaemorrhagiae from the serogroups Canicola and Pomona. *L. santarosai* isolates presented higher genetic variation than the other species in both techniques. Interestingly, the minimum inhibitory concentration (MIC) cluster analysis also provided *Leptospira* serogroup differentiation. Further studies are necessary regarding serovar Bananal isolates, as they presented the highest MIC values for most of the antimicrobials tested. All studied techniques successfully distinguished *Leptospira* species and serogroups. Despite being library-dependent methods, these approaches are less labor intensive and more economically viable, particularly SE-AFLP, and can be implemented in most reference laboratories worldwide to enable faster *Leptospira* typing.

## INTRODUCTION

Leptospirosis is a worldwide systemic zoonosis, with higher incidence in tropical climates.^1^ This disease is caused by bacteria of the *Leptospira* genus, which are classified into 21 species and nearly 300 serovars organized into 29 serogroups. Among these 21 established species, nine are characterized as pathogenic and frequently isolated from humans and animals; five are considered to be intermediately pathogenic with the ability to infect humans and animals, although less frequently and with variable clinical signs; and seven are considered saprophytic environmental non-pathogenic species.^2, 3, 4, 5^

Until now, the species identification level was defined by DNA-DNA hybridization data, whereas *Leptospira* serogroup and serovar classification is based on the expression of the surface antigens.^2^ Further serological identification is complicated because various serovars can be distributed among different species.^6^ Although the cross-agglutinin absorption test (CAAT) is still considered the standard method for *Leptospira* identification, it is highly laborious and expensive because it requires the maintenance of all reference strains and the production of respective antisera.

In this context, molecular methods, with higher discriminatory power and the ability to establish the molecular epidemiology of the isolates and intra-serovar variation, have been applied for *Leptospira* characterization.^3, 7, 8, 9, 10^ Despite the widespread use of sequence-based molecular methods, with low- to high-throughput scales, they require the use of expensive equipment, rigorously standardized sample preparation protocols and complex bioinformatics analysis. Some genotyping methods of restriction patterns can be quicker and easier to perform; digital analysis enables the standardization and more accurate interpretation of band patterns^10^ and is more economically viable for the vast majority of researchers from developing countries.

This study aimed to characterize *Leptospira* spp. isolated from various hosts in Brazil, at different time periods, by single-enzyme amplified fragment length polymorphism (SE-AFLP), pulsed-field gel electrophoresis (PFGE) and broth microdilution for susceptibility profiling, in an attempt to differentiate *Leptospira* species, serogroups and serovars.

## MATERIALS AND METHODS

### Bacterial isolates and culture conditions

A total of 47 *Leptospira* isolates were studied. These isolates originated from the bacterial collection of the Laboratory of Bacterial Zoonosis—University of São Paulo. They were isolated from various hosts, including swine, dog, rat, bovine and human, at different time periods and from different Brazilian states. Forty isolates were previously serotyped at the WHO/FAO/OIE and National Collaborating Centre for Reference and Research on Leptospirosis (Kit Biomedical Research, Amsterdam, the Netherlands) to determine the respective serogroups and serovars.

Cultures were stocked in Fletcher's medium (DIFCO/USA), enriched with 15% rabbit serum and maintained in EMJH medium (DIFCO/USA) at 30 °C until molecular analysis. The *L. interrogans* serogroup Pomona serovar Pomona reference strain 13A (1937, Australia) and *L. interrogans* serogroup Icterohaemorrhagiae serovar Copenhageni strain L1.130 (1996, Brazil) were used in this study as internal and quality-control serovars for the experiments.

### Molecular typing

#### Species identification by 16S rRNA sequencing

The species of the isolates that did not belong to *L. interrogans* were identified by 16S rRNA sequencing. Purified DNA was recovered according to the Boom *et al.*^11^ protocol and stored at −20 °C. The 16S rRNA gene amplification was performed as previously described by Morey *et al.*^12^ with primer modifications (D1mod—GTT TGA TCC TGG CTC AG; P2mod—GGC TAC CTT GTT ACG ACT T). The amplified fragments were purified using a Illustra GFXTM PCR DNA and Gel Band Purification Kit (GE Healthcare, Little Chalfont, UK) according to the manufacturer's protocol and sequenced directly at the Human Genome Research Center (University of São Paulo, Brazil). The BIOEDIT Sequence Alignment Editor 7.0.9 ^13^ was used for sequence editing. The DNA sequences from this study were deposited in GenBank under the accession numbers KJ946433—KJ946437, KP739777—KP739784 and KU053945—KU053947.

#### Single-enzyme amplified fragment length polymorphism (SE-AFLP)

SE-AFLP was performed according to the protocol of McLauchlin *et al.*^14^ DNA fragments were detected by electrophoresis at 24 V for 26 h in 2% agarose gel stained with BlueGreen (LGC Biotecnologia, São Paulo, Brazil), and images were captured under UV illumination by a Gel Doc XR System (Bio-Rad Laboratories, Hercules, CA, USA).

#### Pulsed-field gel electrophoresis (PFGE)

*Leptospira* seven-day cultures were centrifuged at 5000 rpm for 20 min. The supernatant was discarded, and the pellet was resuspended in 10 mL of PETT IV solution (10 mmol/L Tris-HCl [pH 8.0], and 1 mol/L NaCl, 10 mmol/L EDTA). The bacterial suspension was centrifuged at 1500 rpm for 10 min, the supernatant was discarded, and the pellet was suspended in 1 mL of lysis buffer (1 mol/L NaCl, 10 mmol/L Tris [pH 8.0], 200 mmol/L EDTA, 0.5% sarcosyl, and 0.2% sodium deoxycholate). Agarose SeaKem gold 2% (Cambrex Bio Science Rockland Inc., East Rutherford, NJ, USA) was prepared in 0.5 × Tris Borate EDTA buffer. A volume of 400 μL of the bacterial suspension was heated to 40 °C and added to 20 μL of 100 mg of lysozyme/mL (LGC Biotecnologia, São Paulo, Brazil) and 400 μL of heated 2% agarose solution. The mixture was immediately dispensed into wells and chilled for 10 min at 4 °C. Plugs were placed in 2.5 mL of lysis buffer, and 70 μL of proteinase K (20 mg/mL; LGC Biotecnologia) was added before overnight incubation at 56 °C. The plugs were rinsed once in 1 ml Tris EDTA buffer (10 mmol/L Tris, 1 mmol/L EDTA). The plugs were washed twice with 5 mL of Tris EDTA buffer (10 mmol/L Tris, 1 mmol/L EDTA) for 30 min and then stored in one mL of Tris EDTA buffer (10 mmol/L Tris, 1 mmol/L EDTA) at 4 °C.

DNA was cleaved with *Not*I enzyme (New England BioLabs, Ipswich, MA, USA), and the PFGE run was performed as described by Galloway and Levett.^8^ The gels were stained with one(Sybr Safe (Invitrogen Corporation, Carlsbad, CA, USA) for 40 min and photographed under UV transillumination. The DNA fragments were identified using a Lambda DNA-PFGE marker (New England BioLabs, Ipswich, MA, USA), and the images were captured by the Gel Doc XR System (Bio-Rad Laboratories, Hercules, CA, USA).

### Broth microdilution susceptibility testing

The susceptibility profiles of the isolates were determined by the antimicrobial minimum inhibitory concentration (MIC) values obtained using the broth microdilution technique. The broth microdilution method was adapted from Murray and Hospenthal's protocol^15^ for the use of the Sensititre Standard Susceptibility MIC Plate BOPO6F (TREK Diagnostic Systems/Thermo Fisher Scientific, Waltham, MA, USA). For the inoculum, cultures were grown at 30 °C for seven days and diluted to an optical density, at 420 nm, of 0.32 (approximately 10^8^ CFU/mL), followed by serial dilution using EMJH medium to achieve a final concentration of 2 × 10^6^ CFU/mL (the inoculum was also confirmed by enumeration in a Petroff-Hausser chamber under dark-field microscopy). Fifty microliters of the inoculum were distributed to each well of the Sensititre MIC Plate, and after three days of incubation, 5 μL of 10X alamarBlue (Thermo Fisher Scientific, Waltham, MA, USA) was added to each well. The MIC were assessed visually as the lowest concentration of antibiotics in the wells without color change of alamarBlue on the fifth day of incubation. The susceptibility testing was performed once for each isolate.

### Statistical analysis

The phylogenetic analysis of 16S rRNA sequencing was performed using Mega 5.10.^16^ A dendrogram was constructed using the maximum-likelihood method with the Tamura-3-parameter model. The SE-AFLP and PFGE results were analyzed using the Bionumerics 7.5 software (Applied Maths NV, Saint-Martens-Latem, Belgium). Fingerprint patterns were analyzed by a comprehensive pairwise comparison of restriction fragment sizes, using the Dice coefficient. The mean values obtained from Dice coefficients were employed in UPGMA to generate dendrograms. For SE-AFLP, a cut-off value of 90% of genetic similarity was applied to analyze the resulting clusters; for the PFGE analysis, the isolates were considered to belong to different pulsotypes when differing by four or more bands.^17^ The discriminatory indexes of both techniques were calculated as described by Hunter and Gaston.^18^ The MIC cluster analysis was also performed with Bionumerics 7.5 (Applied Maths NV, Saint-Martens-Latem, Belgium) using the Rank correlation method.

## RESULTS

**Figure 1:**
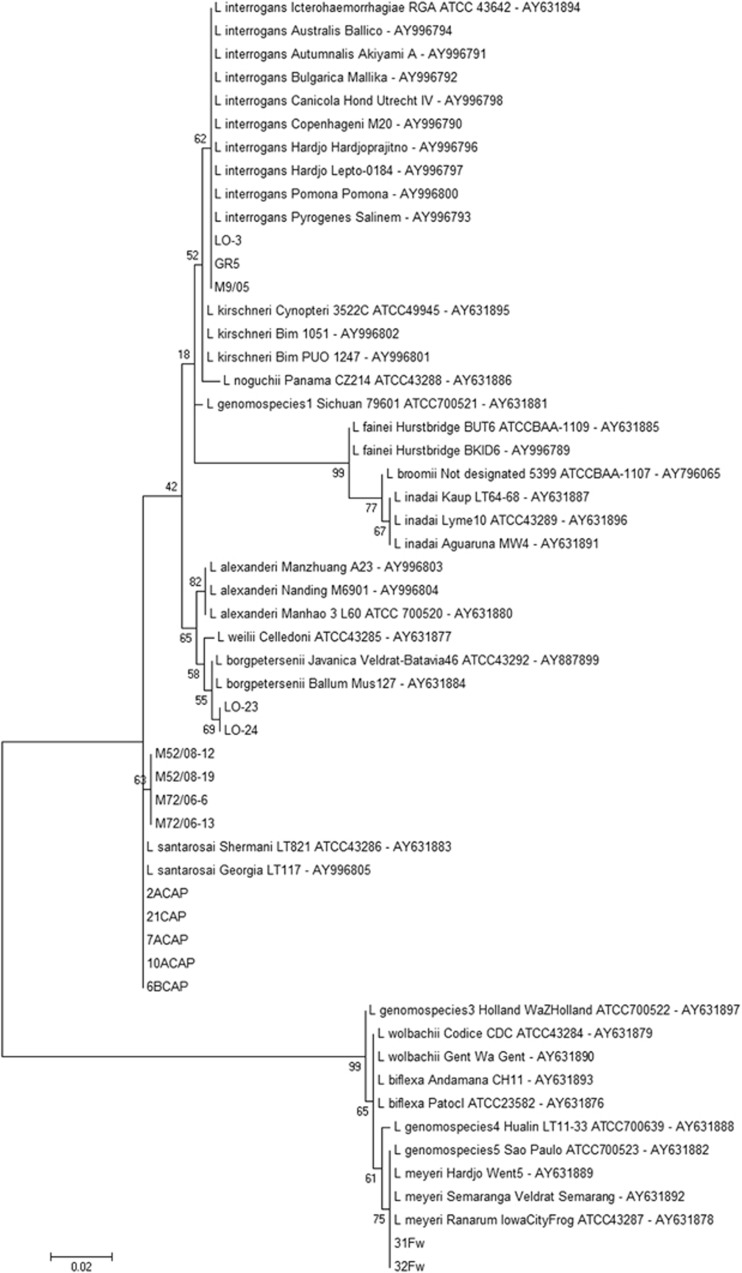
Dendrogram showing the species confirmation of the non-*L. interrogans* isolates based on 16S rRNA nucleotide sequences. The bootstrap values presented at corresponding branches were evaluated using 500 replicates.

**Figure 2:**
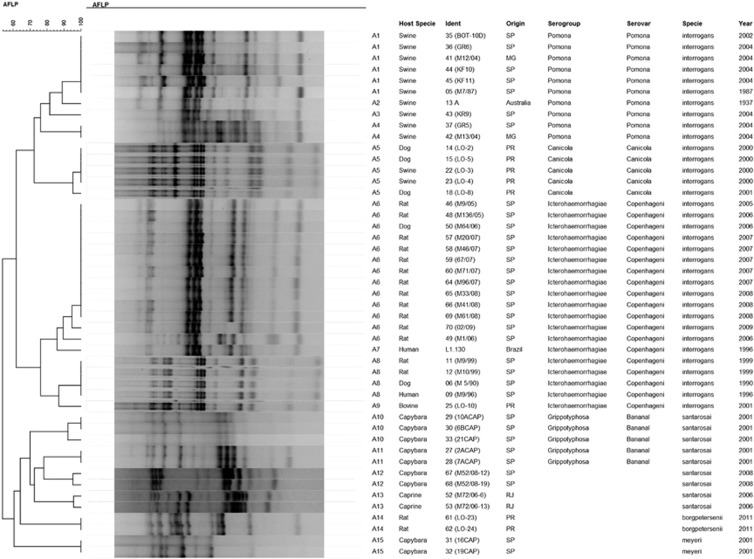
Dendrogram showing the relationships among the SE-AFLP patterns from *Leptospira* spp. isolates.

The *L. santarosai*, *L. meyeri* and *L. borgpetersenii* species were confirmed by 16S rRNA sequencing (Figure 1). SE-AFLP analysis resulted in 15 profiles (A1-A15) comprising the 47 *Leptospira* isolates (Figure 2). This technique enabled the distinction of *L. interrogans* from *L. santarosai*, *L. meyeri* and *L. borgpetersenii* in two main clusters, with over 60% genetic similarity. Among *L. interrogans*, it was possible to differentiate into two new sub-clusters the serogroup Icterohaemorrhagiae from the serogroups Canicola and Pomona. Serogroup Canicola clustered in profile A5, and the 10 isolates of serogroup Pomona were grouped in four profiles (A1-A4). Although the *L. interrogans* serogroup Icterohaemorrhagiae clustered together with more than 70% similarity, the 19 isolates were further differentiated into four profiles (A6-A9).

To distinguish them from the *L. interrogans* cluster, *L. santarosai*, *L. meyeri* and *L. borgpetersenii* isolates could be differentiated from each other at the species level within their cluster. Although the majority of *L. santarosai* were typed as serogroup Grippotyphosa serovar Bananal and clustered together with over 70% similarity, they presented higher genetic variability than the other isolates and were classified into four profiles (A10-A13). There was no apparent relationship between host species, time period and local of isolation with the SE-AFLP profiles for *L. santarosai*. The only two *L. borgpetersenii* isolates were classified as indistinguishable, as were the *L. meyeri* isolates.

**Figure 3:**
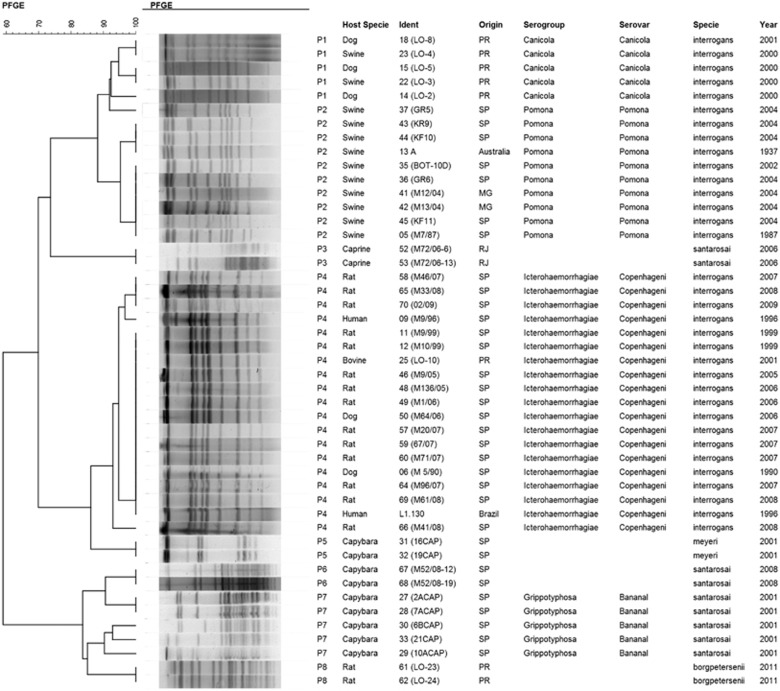
Dendrogram showing the relationships among the *Leptospira* spp. pulsotypes.

Molecular typing with PFGE also presented the tendency to differentiate *L. interrogans* from the other *Leptospira* species studied with more than 60% genetic similarity; however, two *L. santarosai* isolates (M72/06-6 and M72/06–13) and two *L. meyeri* isolates (16CAP and 19CAP) presented pulsotypes more closely related to the *L. interrogans* serogroups Pomona and Icterohaemorrhagiae, respectively (Figure 3). Despite presenting similar results to SE-AFLP with the distinction of *Leptospira* species in two main clusters and a tendency to segregate *L. interrogans* serogroups, PFGE resulted in fewer band patterns with a total of eight pulsotypes (P1-P8) comprising the 47 studied isolates.

Serovar Canicola isolates clustered in P1, whereas P2 corresponded to serovar Pomona isolates and P4 to serogroup Icterohaemorrhagiae. Although PFGE enabled this distinction of *L. interrogans* serogroups, it also clustered the *L. santarosai* isolates M72/06-6 and M72/06–13 and the *L. meyeri* isolates 16CAP and 19CAP (P3 and P5, respectively) in the main group of *L. interrogans*. Similar to the SE-AFLP results, the *L. santarosai* isolates presented higher genetic variation in PFGE analysis than the other *Leptospira* species. The discriminatory indexes obtained for the SE-AFLP and PFGE techniques were 0.89 and 0.78, respectively.

**Figure 4:**
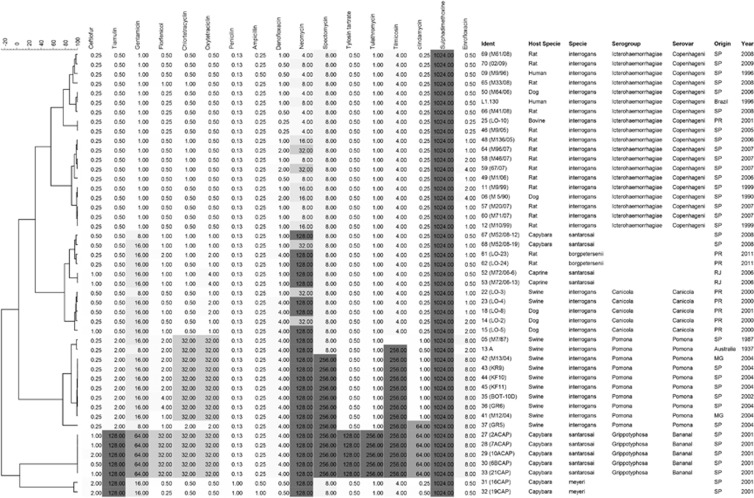
Dendrogram showing the relationships among the antimicrobial susceptibility profiles of *Leptospira* spp. isolates. Gray-scale coloring according to increasing MIC values of tested antimicrobials. MIC values presented correspond to single test results.

The MIC results for susceptibility testing are shown in Figure 4. With the exception of penicillin and ampicillin, to which all isolates were susceptible, and trimethoprim/sulfamethoxazole and sulfadimethoxine, which presented higher MIC values, the other antimicrobials tested presented variable MIC values. Cluster analysis based on MIC values also enabled the differentiation of *Leptospira* serogroups; however, it did not present the same distinction of species as genotyping. Five clusters can be defined in Figure 4 with greater than 50% similarity: the first comprises serogroup Icterohaemorrhagiae isolates with the lowest MIC profile. However, these isolates already present higher values for neomycin, tilmicosin, spectomycin and fluoroquinolones than expected. The increasing MIC values for gentamicin, oxytetracyclin and neomycin determined the second cluster of *L. santarosai* and *L. borgpetersenii* and a subcluster of *L. interrogans* serogroup Canicola isolates with danofloxacin MIC of 4 mg/L.

Elevated MIC values for tiamulin, chlortetracycline, oxytetracyclin, tilcomisin and clindamycin, as well as for danofloxacin, neomycin and spectomycin, characterized the *L. interrogans* serogroup Pomona cluster. The other two clusters comprised the *L. santarosai* serogroup Grippotyphosa serovar Bananal isolates, which presented the most alarming susceptibility profile, with high MIC values for most of the tested antibiotics with the exception of the β-lactams, and *L. meyeri,* which also presented a distinct profile with the only isolates with a ceftiofur MIC value of 2 mg/L.

## DISCUSSION

Molecular techniques have been applied to *Leptospira* characterization in an attempt to differentiate species and serovars; however, most *Leptospira* can be identified only at the species level. Although the practical clinical relevance of serovar identification is still questioned, it is considered an important data source for the study of leptospirosis epidemiology.^8^ As serovars are associated with specific hosts and even disease severity, their identification can enable the prediction of infection sources and directly assist in controlling the spread of the disease.^8, 9, 19^

Our genotyping results, distinguishing *L. santarosai*, *L. borgpetersenii* and *L. interrogans* with greater than 60% genetic similarity, corroborate previous DNA-DNA hybridization data for species definition.^19, 20^ The application of PFGE and SE-AFLP for *Leptospira* species differentiation represents an alternative for gene sequencing and other molecular methods that are limited to certain pathogenic species, such as the variable number of tandem repeats (VNTRs). SE-AFLP also presents the further advantage of being faster, more economically viable and less troublesome than PFGE and presenting similar results.

Regarding *Leptospira* serotyping, even though the serovars and serogroups are important epidemiological data, serovars have already been considered a poor indicator of *Leptospira* genetic relatedness.^3^ To date, serovar variation has been related to lipopolysaccharide (LPS) structure, specifically the O-antigen, and its biosynthesis locus (*rfb* cluster).^21, 22^ As molecular typing methods are not directly related to the *rfb* gene cluster, their application to serovar and serogroup assessment remains controversial.

However, the observed distinction of *L. interrogans* and *L. santarosai* serovars by both PFGE and AFLP also corroborates previous reports,^6, 8, 23^ such that these methods can be considered valuable tools for *Leptospira* typing despite their limitations.^24^ Serogroups must have further genomic differences to allow genotypic distinction. Future studies of comparative genomic analysis may reveal these differences and enhance the molecular serotyping techniques.

Genotyping also enables further epidemiological analysis regarding hosts and infection sources, as we can observe in the SE-AFLP A8 profile that comprises indistinguishable strains of *L. interrogans* serogroup Icterohaemorrhagiae serovar Copenhageni originating from humans, dogs and rats. This information cannot be assessed by other molecular methods, such as multiple locus sequence typing (MLST), which does not present great correlation with serovars, as it can cluster different serovars in the same sequence type, or isolate origins.^8, 9^

Interestingly, the MIC cluster analysis for the susceptibility testing also provided *Leptospira* serogroup differentiation. The few previous studies of *Leptospira* antimicrobial susceptibility focused only on clinical treatment and included a limited number of antimicrobials.^25, 26, 27^ Here, we observed differences among the MIC profiles of *Leptospira* species and of *L. interrogans* serogroups that are relevant for the clinical and epidemiological assessment of leptospirosis.

From the clinical perspective, even though most isolates were susceptible to penicillin and ampicillin, the classical treatment for leptospirosis, the higher MIC values for tetracyclines, fluoroquinolones, aminoglycosides, tiamulin and spectomycin were not expected, and the veterinary usage of these antibiotics requires attention. The variability of fluoroquinolone susceptibility deserves further attention, as norfloxacin has been indicated as an alternative empirical leptospirosis treatment.^28^

Regarding the epidemiological assessment, the MIC profile can serve as an alternative tool for *Leptospira* typing; however, antimicrobial susceptibility is directly related to the host environment and geographical location due to differences in antimicrobial usage worldwide. This relationship could explain the differences between our results and the previous reports by Murray and Hospenthal^26^ and Ressner *et al.*'s,^27^ in which no significant variability was observed among the studied strains' susceptibility profiles. However, further studies are necessary regarding serovar Bananal isolates, which presented the highest MIC values for most antimicrobials, and *L. meyeri,* which presented higher MIC values for the tested β-lactams.

It should be noted that in this study, only 47 *Leptospira* isolates were evaluated, comprising field strains exclusively from one country; the small number of *L. meyeri* and serovar Bananal samples are also drawbacks of the study, despite its noteworthy results. Therefore, larger studies, including isolates from different geographical origins with more representative serogroup/serovar samples, are necessary to allow implementation of the techniques across laboratories worldwide, as well as the assessment of interlaboratory variation.

All three studied techniques have successfully distinguished *Leptospira* species and serogroups. Although they are classified as library-dependent methods and therefore require a database of reference strains for comparative analysis, they are also less troublesome and more economically viable, particularly SE-AFLP, than the recently enhanced sequencing techniques and can be implemented in most reference laboratories worldwide. Respective limitations and geographical variations must be recognized; nevertheless, SE-AFLP, PFGE and susceptibility testing are alternative methods for *Leptospira* typing and enhanced epidemiological analysis.
